# Implementation science at the crossroads

**DOI:** 10.1136/bmjqs-2017-007502

**Published:** 2017-11-28

**Authors:** Richard J Lilford

**Keywords:** patient safety, health services research, implementation science

Martin Marshall and colleagues^1^ take themselves to task for the suboptimal design of a complex (multicomponent) intervention to improve safety of services for people in care homes. The authors make much of the complexity of the intervention—service interventions are ‘not like a pill’. Interventions must be adapted—when first promulgated the intervention in question had nine components, and this inflated to 15 over the course of the project. Contrast all of these with a financial incentive promulgated by the Specialist Services Commissioning authority for the West Midlands, England. Hospitals were simply given a financial incentive to promote a switch from facility to home haemodialysis.^2^

So here we have accounts of what appear to be two very different types of interventions; Marshall’s intervention encapsulates 15 components, while the commissioning agent’s intervention was of one component only. One might think that Marshall’s intervention was complex and the commissioning agent’s was simple. But this is an artefact of how the intervention is conceptualised. The commissioning agent’s incentive could not, by itself, achieve any change. It was necessary for each service to respond to it, to make and then implement a plan. These are described in the paper evaluating the incentive.^2^ These local initiatives are conceptualised as effects of the intervention. These are not the intervention itself. The commissioning agent’s intervention could be conceptualised as a ‘promoter intervention’, since it is designed to promote other interventions ‘downstream’. So the commissioning agent could have promulgated a more complex intervention consisting of the incentive and downstream actions consisting, for example, of a ‘toolkit’ to help implement change. The commissioning agent could have gone further in providing a toolkit  and mentoring on how to implement the toolkit. Similarly, Marshall could have been much less directive—instead of an intervention of 15 components, he could have promulgated the three principles on which his intervention was based and left it up to each care home to come up with their own solution. The point being made here is that how many components an intervention has, and hence how complex it is, is a choice. The system into which an intervention is inoculated is *always* complex, but the intervention may be more or less complex—it may be minimalist or maximalist or somewhere in between. Marshall’s intervention was located towards the maximalist pole, while the commissioning agent’s incentive was minimalist—the detail was left to local providers. So the first corollary that flows from this analysis is the importance of being explicit about the intervention philosophy. An intervention can also be a hybrid, consisting of common elements that all intervention sites are required to implement, and variable elements at local discretion.

Clearly there is no right or wrong answer to the question of how many components should be wrapped up in an intervention. From the perspective of a particular service provider, they should include all the measures taken to maximise the intervention’s chance of success. But the intervention that is promulgated across a health system (or beyond) need not include everything that must be done locally. While accepting that the complexity of the intervention is a matter for judgement—there is no one size fits all—we wish to advocate for a more minimalist approach as a default, and we are somewhat alarmed by the implication in Marshall *et al*’s paper that an intervention as promulgated should seek to embody, by prescription, everything necessary for it to succeed. Our argument for a more minimalist mindset is threefold:

First, since contexts differ, managers need to vary their actions from place to place, just as a cook must improvise in the kitchen. Trying to fix all these different variables in advance may limit room for manoeuvre and may even be demotivating.

Second, attempting a description of an intervention that encompasses every component to be used in practice is a quixotic task; to attempt such a portmanteau description is to set oneself up to fail. We think that ‘less is more’ in many cases.

Third, and perhaps most important, managers do not tend to implement interventions algorithmically—managers follow ‘mindlines’ rather than refer to the intervention manual like a recipe.^3^

In thinking about these matters, it is important to draw a conceptual distinction between an independent evaluation and an intervention done formatively to guide implementation rather than to provide scientific results for general consumption. If the evaluation is part and parcel of the intervention, then the description of the intervention must incorporate that intramural evaluation.^4^ The results can only be generalised insofar as any future implementation incorporates a formative evaluation.

What we need to come up with is interventions that will do more good than harm, without requiring a degree of specification that would be hard to replicate and/or that only works when implemented by a research team committed to the cause. The SQUIRE 2.0 (Standards for Quality Improvement Reporting Excellence) guidelines^5^ set detailed and exacting standards for description of service delivery interventions, which should include a description of how the intervention may have changed over the intervention period. We dispute this principle. The intervention is the set of instructions that can be propagated across a system and beyond. How individual providers interpret and change the intervention should be conceptualised as an *effect* of the intervention as promulgated and should be described as such. Sure, it should be described, just as a chef may explain a change in a recipe or a pianist interprets the music. But the recipe and the score represent the intervention.
